# Supplementary figures and images for: Inhibition of BET Family Proteins Suppresses African Swine Fever Virus Infection
Source: Microbiol Spectr. 2022 Jun 27;10(4):e02419-21. doi: 10.1128/spectrum.02419-21 (PMC9430462; doi:10.1128/spectrum.02419-21)

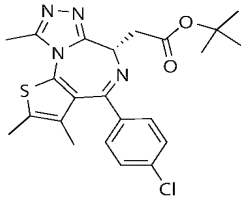

(+)-JQ1

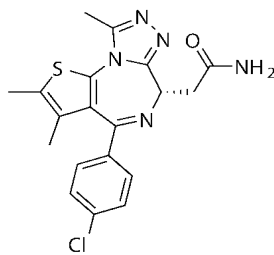

CPI-203

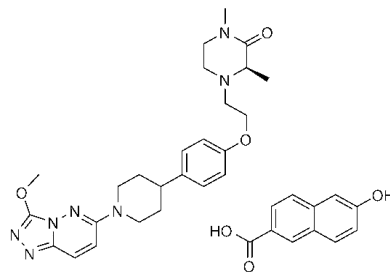

AZD5153

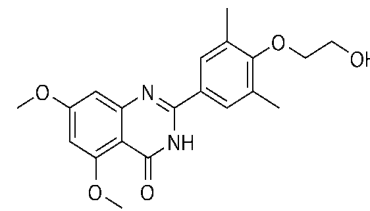

RVX-208

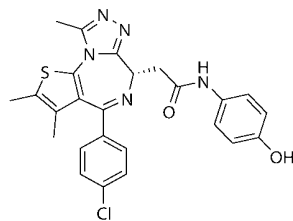

OTX051

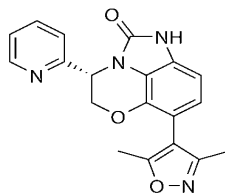

INCB054329

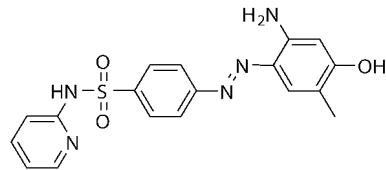

MS436

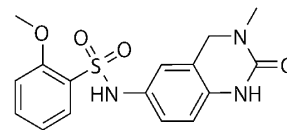

PFI-1

Figure S1. Structure of BET/BRD4 inhibitors

Supplement: Supplemental file 1 — Supplemental material. Download spectrum.02419-21-s0001.pdf, PDF file, 0.2 MB [file spectrum.02419-21-s0001.pdf]
